# *H2AFZ*: A Novel Prognostic Marker in Canine Melanoma and a Predictive Marker for Resistance to CDK4/6 Inhibitor Treatment

**DOI:** 10.3389/fvets.2021.705359

**Published:** 2021-08-16

**Authors:** Laura Bongiovanni, Anneloes Andriessen, Serenella Silvestri, Ilaria Porcellato, Chiara Brachelente, Alain de Bruin

**Affiliations:** ^1^Department of Biomolecular Health Sciences, Faculty of Veterinary Medicine, Utrecht University, Utrecht, Netherlands; ^2^Department of Veterinary Medicine, University of Perugia, Perugia, Italy; ^3^Department of Pediatrics, University Medical Center Groningen, University of Groningen, Groningen, Netherlands

**Keywords:** dog, melanoma, cancer biomarker, CDK4/6 inhibitor, E2F target genes

## Abstract

Uncontrolled proliferation is a key feature of tumor progression and malignancy. This suggests that cell-cycle related factors could be exploited as cancer biomarkers and that pathways specifically involved in the cell cycle, such as the Rb-E2F pathway, could be targeted as an effective anti-tumor therapy. We investigated 34 formalin-fixed paraffin-embedded (FFPE) tissue samples of canine cutaneous melanocytoma, cutaneous melanoma, and oral melanoma. Corresponding clinical follow-up data were used to determine the prognostic value of the mRNA expression levels of several cell cycle regulated E2F target genes (E2F1, DHFR, CDC6, ATAD2, MCM2, H2AFZ, GINS2, and survivin/BIRC5). Moreover, using four canine melanoma cell lines, we explored the possibility of blocking the Rb-E2F pathway by using a CDK4/6 inhibitor (Palbociclib) as a potential anti-cancer therapy. We investigated the expression levels of the same E2F target gene transcripts before and after treatment to determine the potential utility of these molecules as predictive markers. The E2F target gene H2AFZ was expressed in 91.43% of the primary tumors and H2AFZ expression was significantly higher in cases with unfavorable clinical outcome. Among the other tested genes, survivin/BIRC5 showed as well-promising results as a prognostic marker in canine melanoma. Three of the four tested melanoma cell lines were sensitive to the CDK4/6 inhibitor. The resistant cell line displayed higher expression levels of H2AFZ before treatment compared to the CDK4/6 inhibitor-sensitive cell lines. The present results suggest that CDK4/6 inhibitors could potentially be used as a new anti-cancer treatment for canine melanoma and that H2AFZ could serve as a prognostic and predictive marker for patient selection.

## Introduction

Increased proliferation is an important characteristic of tumorigenesis. The proliferation rate is routinely estimated immunohistochemically using the Ki67 antibody (MIB-1). The level of Ki67 expression (as Ki67 index) is widely accepted as an indicator of prognosis and used as a prognostic marker in a number of human (1) and canine cancers (2–6). This indicates that a high degree of proliferation is a common feature of tumor malignancies and their progression, suggesting the potential to target molecules and pathways specifically involved in cell proliferation and cell cycle as an effective anti-tumor therapy (7).

One of the most often deregulated pathways during cancer development and progression is the Rb-E2F pathway. This pathway is initiated by cyclin-dependent kinases (CDKs) that are able to phosphorylate the Rb protein. Once Rb is hyperphosphorylated, it detaches from the E2F proteins. E2F family members are transcription factors playing a crucial role in the regulation of cellular proliferation, apoptosis, and differentiation. The E2F family consists of activator E2Fs and repressor E2Fs that together provide a balanced regulation of the expression of E2F transcriptional target genes (8). E2F target genes are involved in cell cycle regulation, DNA replication, DNA repair, and mitosis (9), thus, their transcripts seem to represent valuable prognostic biomarkers in cancer. Indeed, to date several E2F target genes have been proposed as tissue prognostic markers in different human tumor types, including non-small cell lung cancer, lymphoblastic leukemia, and gastric cancer (10–16). However, data on E2F target genes in canine cancers are extremely limited. Survivin, a protein encoded by the E2F target gene *BIRC5*, has been suggested as a prognostic marker in several canine tumors, including cutaneous melanoma (17).

In dogs, melanocytic tumors can arise from the skin and from the mucosa, with the oral mucosa being the most frequent location of this type. While cutaneous melanocytic tumors in dog are frequently bening (called melanocytoma), canine oral malignant melanoma (MM) is in general a highly aggressive tumor, with high similarities with the human counterpart (18–20). Similar to human mucosal MM, canine oral MM is often associated with an aggressive malignant behavior with rapid invasion of surrounding normal tissues, frequent metastasis (21), and resistance to therapy (22).

Although numerous works have aimed to find new effective prognostic markers for canine MM, there is still a lack of reliable ones, and Ki67, beside specific histological features of malignancy, remains the only currently available established prognostic marker (2, 23, 24). However, there are still some limitations to its use, such as the presence of some cases with low Ki67 index but poor prognosis (2), the lack of consistency in Ki67 assessment on microscope, and inter-observer variations (25).

CDK4/6 inhibitors, namely palbociclib, ribociclib, and abemaciclib, represent novel effective therapies and are currently Food and Drug Administration (FDA) and European Medicines Agency (EMA) approved for the treatment of breast cancers in humans, in combination with other therapeutics (26, 27). They share the same mechanism of action-based on the specific inhibition of the Rb-E2F pathway and consequent cell cycle arrest. A growing body of evidence indicates that these drugs could be used for other malignancies, such as those with poor prognoses, including metastatic melanoma (27). Cyclin-dependent kinases CDK4 and CDK6 represent promising therapeutic targets in human melanoma. Their inhibition affects cancer cell proliferation, blocking the progression from G1 to S phase, and inhibit the metastatic potential of melanoma cells, reducing their migration and angiogenesis (28). Furthermore, CDK4/6 inhibition is able to induce anti-tumor immunity by increasing tumor immunogenicity. This effect seems to be mediated by different mechanisms: enhancing tumor antigen presentation, suppressing the proliferation of regulatory T cells (Tregs) and, as a consequence, promoting cytotoxic T cell-mediated clearance of tumor cells (29). Specifically, the anti-proliferative effects of CDK4/6 inhibitors on both tumor cells and Tregs appear to be associated with reduced activity of DNA methyltransferase 1, that is encoded by the E2F target gene *DNMT1* (29). In dogs, CDK4/6 inhibitors have been recently proposed for the treatment of canine mammary tumors, based on promising *in vitro* investigations (30).

In order to select patients for this specific type of therapy, biomarkers are required. Loss of function of the Rb protein, as well as Cyclin E and E2F3 gene amplification have been identified as new putative markers of CDK4/6 inhibitor resistance (31). CDK4, CDK6, and cyclin D1 amplification, and CDKN2A loss are considered markers of CDK4/6 inhibitor sensitivity (31). Since the Rb-E2F pathway ends with the activation of E2F target genes, their expression levels could be investigated as potential sensitivity or resistance markers of CDK4/6 inhibition.

The aim of the present work was to investigate the prognostic utility of a pool of E2F target genes by correlating their levels of expression in primary melanocytic tumors with clinical follow-up data. Secondly, we wanted to investigate the effect of a CDK4/6 inhibitor in four oral melanoma cell lines to provide a valid scientific rationale for the use of this approach in the treatment of canine oral melanoma. Finally, the variations of E2F target gene expression in treated and untreated, resistant or sensitive cell lines were analyzed in order to understand the potential utility of these molecules as predictive biomarkers.

## Materials and Methods

### Patients' Selection, Follow Up Data Collection

A retrospective study was performed on formalin-fixed, paraffin-embedded (FFPE) tissue samples submitted to the diagnostic laboratory or veterinary teaching hospitals of the Department of Veterinary Medicine of the University of Perugia in the period between 2009 and 2016. Cases were included in the present study only if they met the following inclusion criteria:

- good quality of the specimen (i.e., no artifacts due to incomplete fixation or fulguration artifacts);
- diagnosis of primary mucosal melanoma, cutaneous melanoma, or cutaneous melanocytoma, confirmed by immunohistochemistry (positivity for either Melan A or PNL2) for poorly pigmented or amelanotic cases;
- no antineoplastic therapy (i.e., chemotherapy, radiation) before surgery/biopsy;
- for incisional biopsies of large tumors, a surface area on cut section >1 cm;
- availability of follow-up information and a minimum follow-up period of 365 days after surgery.

From our sample series tumor relapses and metastases were excluded.

Thirty-four melanocytic tumors (14 oral melanomas, 16 cutaneous melanomas, 4 cutaneous melanocytomas) were selected. Follow-up information was collected for each case: clinicopathological information was retrieved through telephonic interviews with referring veterinarians or through the collection of medical records data from internal cases (Supplementary Table 1). The clinical outcome of dogs that died because of the tumor was considered “unfavourable,” while the one of patients that survived or died due to causes unrelated to melanoma was considered “favourable.” The overall survival (OS) was the time from first diagnosis/appearance to death for any cause; disease-free survival was the time from first diagnosis/appearance to the first event of recurrent disease or metastasis or death. The recurrence was considered as the reappearance of the tumor at the site of origin after removal.

### Tumor Tissue Collection and Histologic Examination

Samples in this study were partially included in a previous study on the usefulness of tumor thickness and modified Clark level for the evaluation of canine melanocytic tumors (24). All samples were histologically evaluated for the parameters having the greater validity for prognostic use in canine melanocytic neoplasia according to the current literature (23, 24).

Sections (4 μm thick) were cut from the paraffin blocks and stained with H&E. The tumors were evaluated blindly and independently by two boarded pathologists (CB, IP). Tumors were reclassified using histologic parameters with the highest level of statistically supported validity for prognostic use in canine melanocytic neoplasia, based on the recent classification (23, 24). According to these criteria, all tumors that had concomitant lymph node/distant metastases had been correctly classified as malignant (“melanoma”). Mitotic count was performed for each case counting the number of mitotic figures in 10 contiguous non-overlapping HPF (40X objective and ocular of 22 mm), starting from the area of highest mitotic activity and avoiding areas with necrosis or severe inflammation. Poorly pigmented, amelanotic melanoma cases were confirmed by immunohistochemistry using anti-MelanA (mouse monoclonal, A103-M27C10-M29E3, Abcam, ab200544, antigen retrieval in Tris-EDTA pH 9.0, dilution 1:150) and anti-PNL2 antibodies (mouse monoclonal, Santa Cruz, sc-59306, antigen retrieval in Citrate buffer pH 6.0, dilution 1:150). Ki67 index was evaluated on slides stained by immunohistochemistry using anti-ki67 antibody (clone MIB-1, Code number: GA626, Agilent-Dako, Glostrup, Denmark, antigen retrieval in Tris-EDTA pH 9.0, dilution 1:150), and calculated by means of image analysis on a minimum of five photos (40x), acquired from intratumoural hotspots. Areas of necrosis, inflammation, and superficial ulceration were avoided. Pictures were taken from non-contiguous hot spot areas where Ki-67 seemed to be highly expressed. The minimum number of cells/cases to count was set to 1,000. The count was performed manually by one operator using the Multi-point tool of ImageJ software (NIH, Bethesda, MD, USA). Ki67 index was calculated as the number of positive cells on the total number of counted cells. Negative and positive controls (Supplementary Figure 1) were applied in the immunohistochemical experiments for the three antibodies.

### Cell Lines

Four canine melanoma cell lines were used (Supplementary Table 2): LMCK, CMM10, and CMM12 were kindly provided by N. Sasaki, T. Nakagawa, K. Saeki, University of Tokyo, Japan (32); OLGA by Raffaella de Maria, University of Turin, Italy (33). The cell lines had been regularly tested and confirmed to be mycoplasma-free. LMCK, CMM10, and CMM12 had been in culture for >70 passages at the time of this study, and OLGA cells were at passage 20. LMCK, CMM10, and CMM12 cell lines were maintained in Dulbecco's Modified Eagle Medium/Ham's F-12 (DMEM/F-12, Invitrogen, Waltham, USA) supplemented with 10% fetal bovine serum (FBS, Invitrogen) and 1% penicillin/streptomycin (P/S, Invitrogen). OLGA cells were maintained in Dulbecco's Modified Eagle Medium (DMEM, Invitrogen) supplemented with 10% FBS and 1% P/S. The cells were cultured in a humidified atmosphere containing 5% CO_2_ at 37°C. All cell lines grew as monolayer cultures and were maintained by passage when they reached over 90% confluence.

### Genes and Primers Selection

To identify candidate biomarkers, we analyzed previously published gene expression signature data from tumor cell lines and tumor tissue samples (34–38) for E2F target genes that are highly expressed in tumors. The most abundantly expressed E2F target genes, with prognostic significance and that had been validated as classical E2F target genes using ChIP-sequencing (39) were selected: *E2F1, DHFR, CDC6, ATAD2, MCM2, H2AFZ, GINS2*, and survivin/BIRC5.

Canine primers for E2F target gene transcripts (Supplementary Table 3) were designed using the Primer3 design tool and were based on coding mRNA sequences. Canine primers for housekeeping genes GAPDH and RPS5 (Supplementary Table 3) were kindly provided by Louis Penning (Utrecht University, the Netherlands).

### RNA Isolation and RT-qPCR

In order to isolate RNA from cells, cells pellets were lysed in RLT buffer (included in the RNeasy mini kit, Qiagen) containing 10% ß-Mercaptoethanol (Merck). RNA was isolated from cells using the RNeasy mini kit (Qiagen, cat. n. 74104) according to the manufacturer's procedure. The optional DNase digestion step was also included.

In order to isolate RNA from FFPE tumor tissue samples, five, 5 μm-thick paraffin sections were obtained for each sample. An RNA isolation commercial kit was used following the manufacturer's instructions (Purelink FFPE Total RNA Isolation Kit, Invitrogen, cat. n. K1621), where at first a deparaffinization step was included. Briefly, 300 μl Melting Buffer was added to FFPE samples, then the samples were centrifuged for 10–20 s at maximum speed (13,000 g) and incubated for 10 min at 72°C. Next, samples were incubated with 20 μl Proteinase K (20 mg/ml) with occasional mixing for 3 h at 60°C directly followed by centrifugation for 1 min at maximum speed. The lysate below the formed thin paraffin layer was transferred into clean RNase-free tubes for the RNA isolation step. Both tissue and cellular RNA quantity and quality were assessed with a Nanodrop® ND-1000 spectrophotometer.

In order to perform reverse transcription and quantitative real-time PCR, cDNA was synthesized using the RevertAid First Strand cDNA Synthesis Kit (Invitrogen) with random hexameric primers. In the qPCR reactions, an equivalent of 10 ng RNA was used with 3 μl 1.5 μM primermix (Biolegio, Nijmegen, the Netherlands), containing both forward and reverse primers, and 12.5 μl SYBR® Green mastermix (Bio-Rad, cat. n. 4364346, California, USA) in a 25 μl reaction. For all primer sets, optimum melting temperatures were determined. Additionally, PCR amplification efficiencies were tested using 10-fold dilution series of cDNA.

Using the BioRad CFX Connect real-time PCR detection system (Bio-Rad, cat. n. 1855201), an initial denaturation step was performed at 95°C for 3 min, followed by 40 cycles of denaturation at 95°C for 10 s, and annealing/extension at 61°C for 30 s. All reactions were performed in duplicate and negative controls were included.

The expression of each E2F target gene was normalized to two reference genes (GAPDH and RPS5). For RNA derived from the cultured cells the normalized expression was presented relative to the expression level of the same genes in the control (untreated) samples [ΔΔCt method, (40)]. For FFPE tumor tissue RNA the normalized expression was presented relative to the expression level of the same genes in the melanocytoma (benign tumor) samples.

### CDK4/6 Inhibitor Treatment

In order to determine the effective dose of CDK4/6 inhibitor palbociclib (PD-0332991, Merck) on canine melanoma cell lines, 1.5 × 10^5^ CMM10, CMM12, LMCK, and OLGA cells were first seeded on p60 dishes in regular cell culture medium without the drug. After 24 h, cell culture media were refreshed and indicated concentrations of the compound (1, 2.5, 5, and 10 μM; dissolved in 1% DMSO) were added. Our group has previously used Palbociclib in RPE cells, where a G1 phase arrest was induced using 1 μM Palbociclib (41). Since the effect of Palbociclib has not yet been investigated in CMM10, CMM12, LMCK, and OLGA cell lines, we used 1 μM as the starting dose to assess whether the canine melanoma cell lines were sensitive to Palbociclib treatment. Control cells were treated with 1% DMSO or received no treatment at all. Cells were harvested after 48 h of treatment to perform cell counts, flow cytometry, and RNA isolation. Cells were counted using a TC20TM automated cell counter.

For FACS analysis, cells were harvested, fixed in 70% ethanol and stored at 4°C for at least 24 h prior to DNA staining.

In the experiments for the evaluation of E2F target genes expression before and after CDK4/6 inhibitor treatment, p60 petri dishes were seeded with 1.5 × 10^5^ cells for OLGA and 2.0 × 10^5^ cells for LMCK, CMM10, and CMM12 in regular cell culture medium. After 24 h, cell culture media were removed and replaced with media containing 1 μM palbociclib dissolved in DMSO. For each cell line, an equal number of untreated cells was used as a control. After 48 h, cells were harvested and used for RNA isolation, cDNA synthesis, and quantification of E2F target gene transcripts by qPCR, as described in the sections “RNA isolation and RT-qPCR.”

All the experiments were carried out in triplicates.

### FACS Analysis

To enable flow cytometric analysis of DNA content, ethanol-fixed cells were mixed with a staining solution containing 2 μg/ml propidium iodide, 500 μg/ml RNase A, and 0.1% bovine serum albumin. Samples were analyzed on a BD FACSCanto II (Becton Dickinson) flow cytometer within 24 h after staining. Flow cytometry data were analyzed using FlowJo software.

### Statistical Analysis

Data relative to gene expression were expressed as mean ± standard deviation (SD). For non-normally distributed variables, median, and interquartile range (IQR) were used. To test for equality of variances between groups, we used *F*-test or Levene's test. Depending on homoschedasticity or heteroscedasticity, we used the Student's *t*-test or Welch test, respectively, to perform comparisons. To compare cell growth between control and treated groups in *in vitro* experiments, we performed ANOVA followed by Dunnett's test with free-step down resampling. To compare non-normally distributed numeric variables, Kruskal-Wallis or Mann–Whitney U-test and Spearman correlation coefficient (ρ) were used. Rho (ρ) was interpreted according to Mukaka (42). For multiple comparisons, we reported Bonferroni-adjusted *P*-values. We performed the univariable Cox proportional-hazards regression to evaluate the association of the expression of the investigated gene transcripts with hazard of death and of developing recurrence/metastasis; results were reported as hazard ratio (HR) with the corresponding 95% confidence interval (95%CI). Statistical analysis was performed with the software R (R version 3.5.2); *P*-values ≤ 0.05 were considered statistically significant.

## Results

### E2F Target Genes H2AFZ and Survivin/BIRC5 Are More Highly Expressed in Melanomas With Unfavorable Clinical Outcome

**Figure 1:**
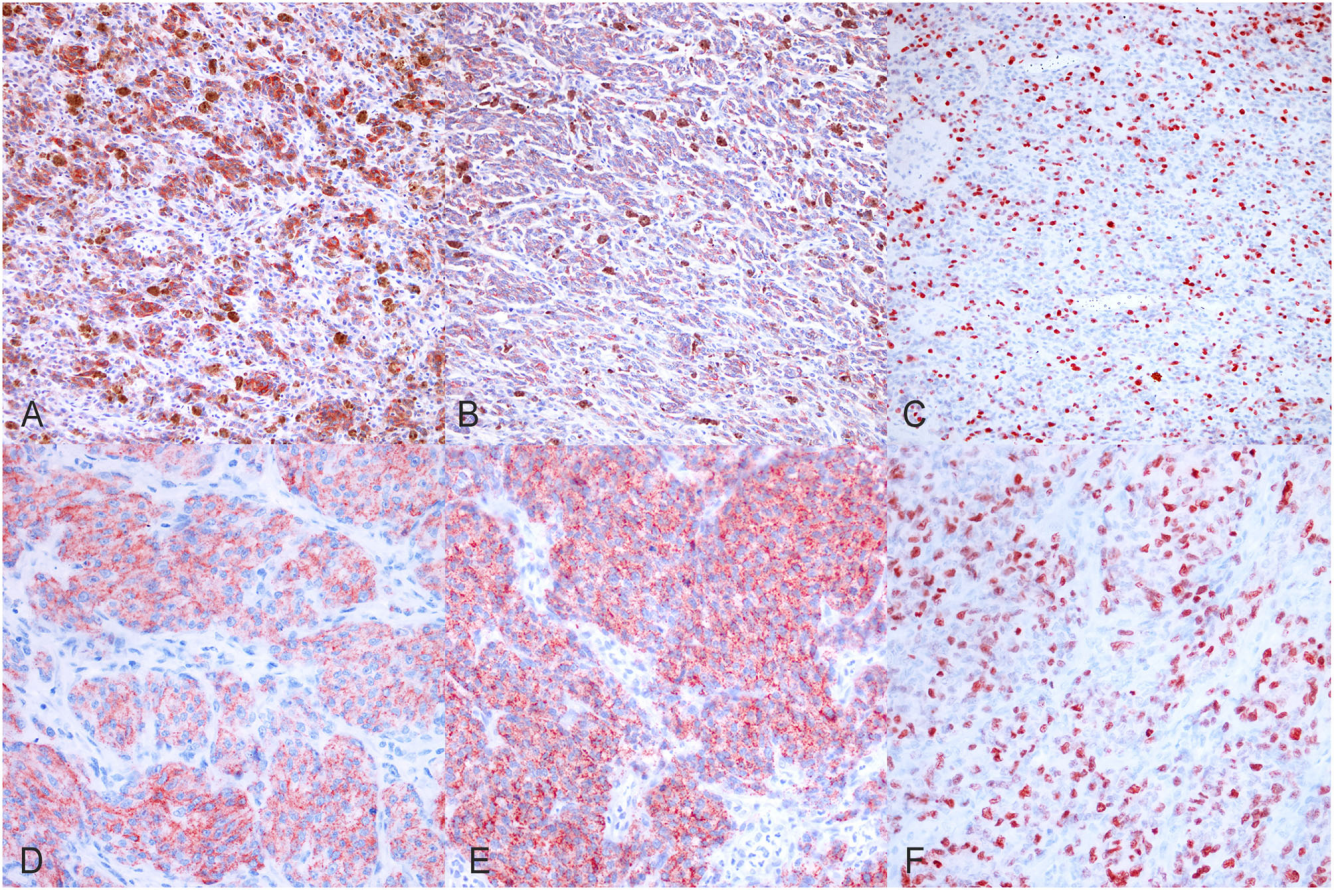
Immunohistochemical results. **(A)** Canine, Oral melanoma (palate). Multifocal, moderate, finely granular, cytoplasmic Melan A immunolabeling (200x, AEC and hematoxylin). **(B)** Canine, Oral melanoma (palate). Multifocal, moderate, finely granular, cytoplasmic PNL2 immunolabeling (200x, AEC and hematoxylin). **(C)** Canine, Oral melanoma (palate). Nuclear immunolabeling of Ki-67 (200x, AEC and hematoxylin). **(D)** Canine, Oral melanoma (gingiva). Diffuse, finely granular, marked, cytoplasmic Melan A immunolabeling (400x, AEC and hematoxylin). **(E)** Canine, Oral melanoma (gingiva). Diffuse, finely granular, marked, cytoplasmic PNL2 immunolabeling (400x, AEC and hematoxylin). **(F)** Canine, Oral melanoma (gingiva). Nuclear immunolabeling of Ki-67 (400x, AEC and hematoxylin).

Before including all the selected canine melanoma cases in the study, we confirmed the melanocytic origin of the neoplastic cells of the poorly pigmented, amelanotic melanoma cases by immunohistochemistry using anti-MelanA and anti-PNL2 antibodies. In the tested cases, we observed multifocal, moderate to marked, finely granular, cytoplasmic Melan A (Figures 1A,D), and PNL2 (Figures 1B,E) immunolabeling.

**Figure 2:**
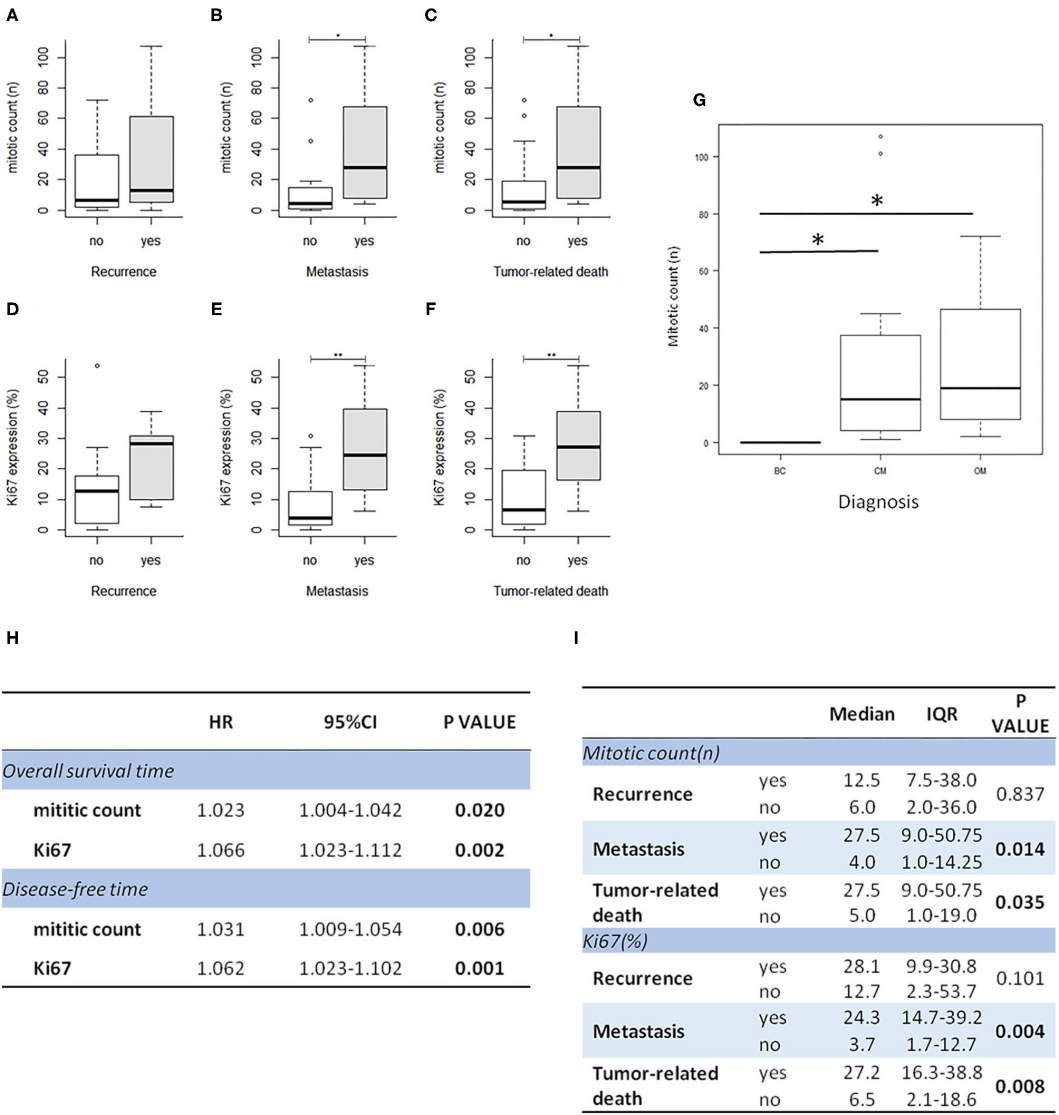
Mitotic count and Ki67 index are higher in melanoma cases with recurrence, metastasis, and unfavorable outcome. Correlation of Ki67 index and mitotic count with clinical follow up data **(A–F)**; correlation of mitotic count with diagnosis (BC, benign cutaneous melanoma; CM, cutaneous malignant melanoma; OM, oral melanoma); *P < 0.05 **(G). (H)** The table shows the results of survival analysis of mitotic count and Ki67 (Cox proportional regression analysis; HR: hazard ratio). **(I)** the table shows the association between mitotic count and Ki67 expression with clinical data (IQR: interquartile range).

In order to investigate the prognostic significance of the selected E2F target genes in canine melanoma samples, we analyzed RNA obtained from FFPE tumor samples of different types of canine melanocytic tumors (melanocytoma, cutaneous, or oral MM) and we compared different groups of patients, considering the following signs of tumor malignancy and progression: mitotic count, presence of recurrence, metastasis, death due to melanoma, disease free interval (DF), OS. Since cutaneous and oral melanoma normally have different clinical behavior, we first investigated the association of diagnosis with malignancy indicators (recurrence, metastasis, and death due to melanoma) as well as with OS time and disease-free time. Additionally, we compared the expression of all genes of interest among the three group. Since no relevant results emerged in our sample population, we considered the diagnosis not influent for the purposes of our study and performed the analysis grouping all tumors (Supplementary Figures 2A–C). Levels of expression of the investigated genes were also correlated with mitotic count and Ki67 index evaluated on immunohistochemical stained slides (Figures 1C,F). Mitotic count and Ki67 index were both significantly higher in cases with metastasis and unfavorable clinical outcome; in line with previous studies, they were associated with diagnosis and an increased risk of death or to develop recurrence/metastasis (Figures 2A–I).

Gene expression was normalized to the level of reference genes GAPDH and RPS5 and fold change expression of the PCR product of each gene was calculated using the mean value of benign melanocytoma cases as reference, based on the ΔΔCt method (40). Six cases (two melanocytoma, three cutaneous, and one oral melanoma cases), in which all genes, including reference genes, were not detectable, were excluded from the analyses.

**Figure 3:**
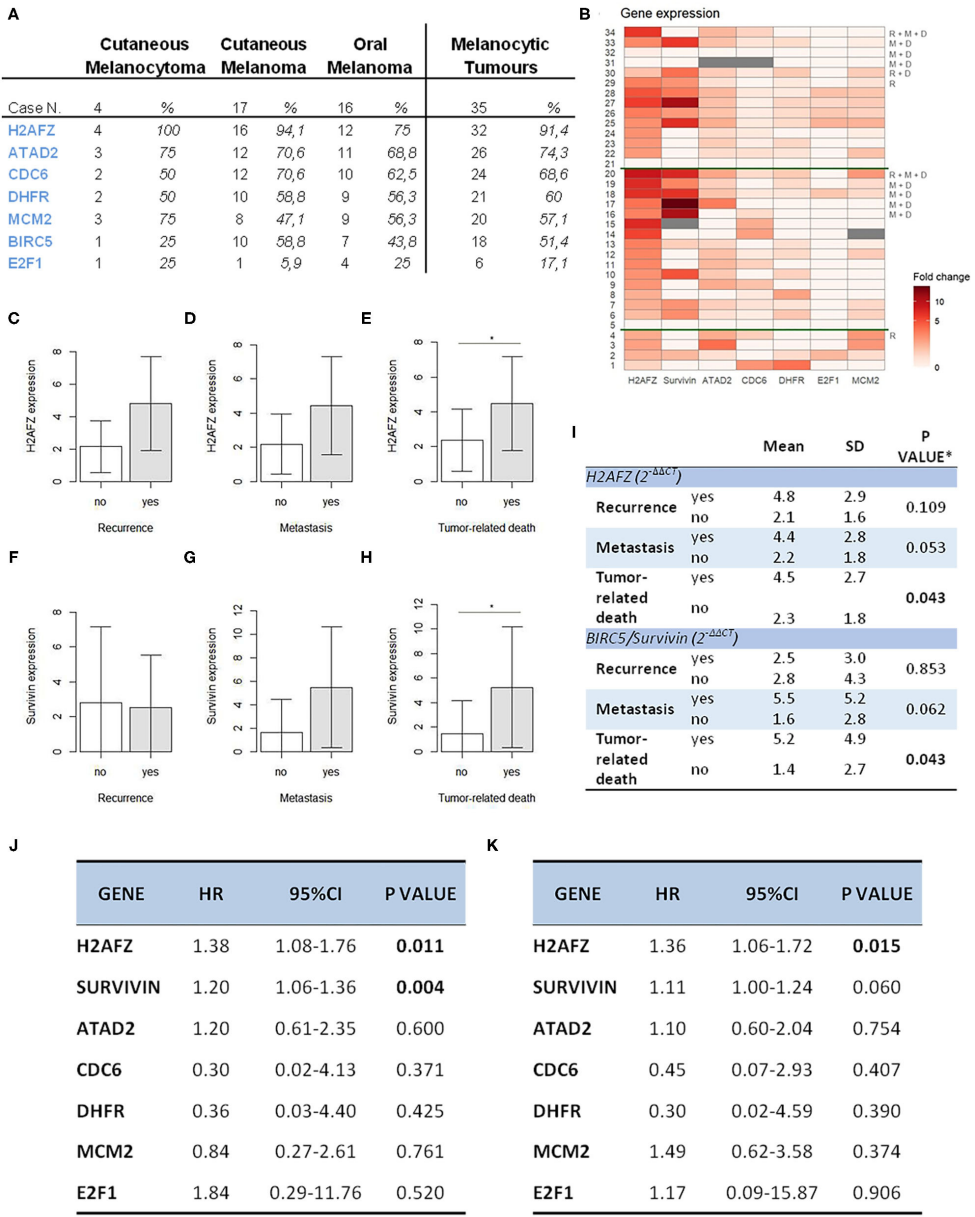
H2AFZ and survivin/BIRC5 mRNA expressions represent prognostic factors in canine melanocytic tumors. **(A)** Table indicating number and percentage of positive cases for all genes in all the melanocytic tumors (right columns) and on specific types. **(B)** Heat map showing gene expression levels for all genes ordered from the bottom to the top: diagnosis (1–4, cutaneous melanocytomas; 5–20, cutaneous melanomas; 21–34, oral melanomas); R, recurrence, M, metastasis, D, tumor-related death; the heatmap was generated by a square root transformation of RT-qPCR data presented as fold change (ΔΔCt method). **(C–H)** Comparison of H2AFZ **(C–E)** and survivin/BIRC5 **(F–H)** transcript expression levels between patient groups. Groups are based on clinical follow-data on recurrence, metastasis, death due to melanoma (unfavorable outcome). Error bars show SDs for each group. *P < 0.05. **(I)** Table shows the association between H2AFZ and survivin/BIRC5 expression with clinical data. *t-test or Welch test, depending on homogeneity of variances. **(J)** Table shows the results of survival analysis of gene expression depending on overall survival time (Cox proportional regression analysis). **(K)** Table shows the results of survival analysis of gene expression depending on disease-free time (Cox proportional regression analysis).

From the data analysis (Figures 3A,B), *H2AFZ* was the most frequently and highly expressed gene in all three groups of melanoma cases, with the highest levels of expression in the cutaneous melanoma.

**Figure 4:**
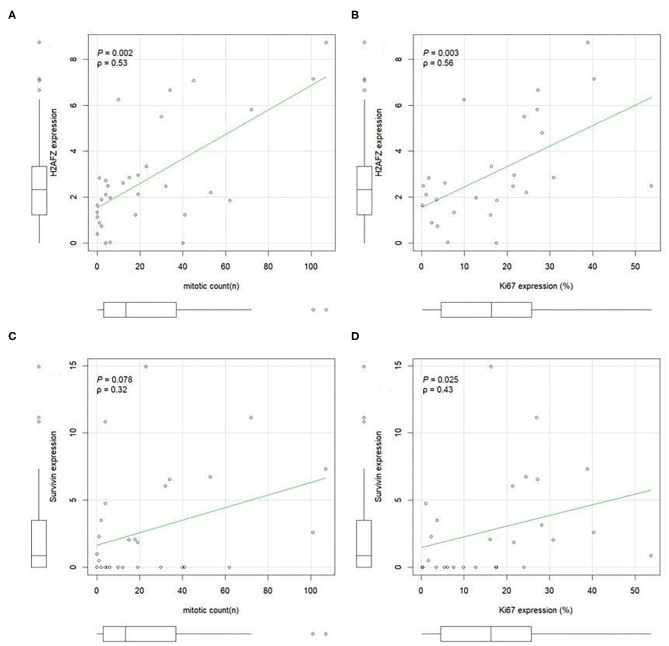
H2AFZ and survivin/BIRC5 mRNA expressions in tumor tissue correlate with histological proliferation markers. A positive correlation was evident between H2AFZ **(A)**, but not survivin/BIRC5 **(C)**, expression levels and mitotic count (MI); while both gene expression levels positively correlated with Ki67 index **(B,D)**. Spearman correlation coefficient. Ki67 was calculated as the number of positive cells on the total number of counted cells (mean 1,090 cells, ranging from 900 to 1,376 cells) using Image J analysis system.

*H2AFZ* mRNA was detectable in 91% of the total cases (Figure 3A), and in all, except one (94.7%) of the cutaneous cases (both benign and malignant). The levels of H2AFZ expression were higher in cases with recurrence (Figure 3C) or metastasis (Figure 3D), but no statistically significant association was found. However, *H2AFZ* expression was significantly higher in cases with unfavorable clinical outcome compared to cases with favorable clinical outcome (Figure 3E). Additionally, *H2AFZ* expression showed a significant moderate positive correlation with the mitotic count (Figure 4A) and the Ki67 index (Figure 4B), and it showed a significant association with increased hazard of death and with the development of recurrence/metastasis (*P* < 0.05) (Figures 3I–K).

Although survivin*/BIRC5* mRNA was expressed at low levels in these samples (Figure 3B), resulting in a lower percentage of cases expressing this gene (51.43%, 18/35) (Figure 3A), differences were observed comparing groups with or without metastasis (Figure 3F), and a significant higher expression was seen in cases with unfavorable clinical outcome compared to those with favorable clinical outcome (Figure 3H). Furthermore, survivin/BIRC5 was significantly associated with increased hazard of death (*P* < 0.01) (Figures 3I–K). A significant low correlation was observed between survivin/BIRC5 gene expression and Ki67 index (Figures 4C,D).

All the other investigated genes were expressed in similar percentages of cases (Figure 3A), but with lower expression compared to H2AFZ and survivin/BIRC5 (Figure 3B). No statistically relevant differences in their expression were found comparing the different groups of patients, nor associations with mitotic count, hazard of developing recurrence/metastasis or death (Supplementary Figure 3).

Data obtained in the present work demonstrate a prognostic significance of *H2AFZ* and survivin/BIRC5 in the cohort of investigated samples of canine melanocytic tumors.

### LMCK, OLGA, and CMM10 Melanoma Cells Are Sensitive, While CMM12 Melanoma Cells Are Resistant to Treatment With a CDK4/6 Inhibitor

**Figure 5:**
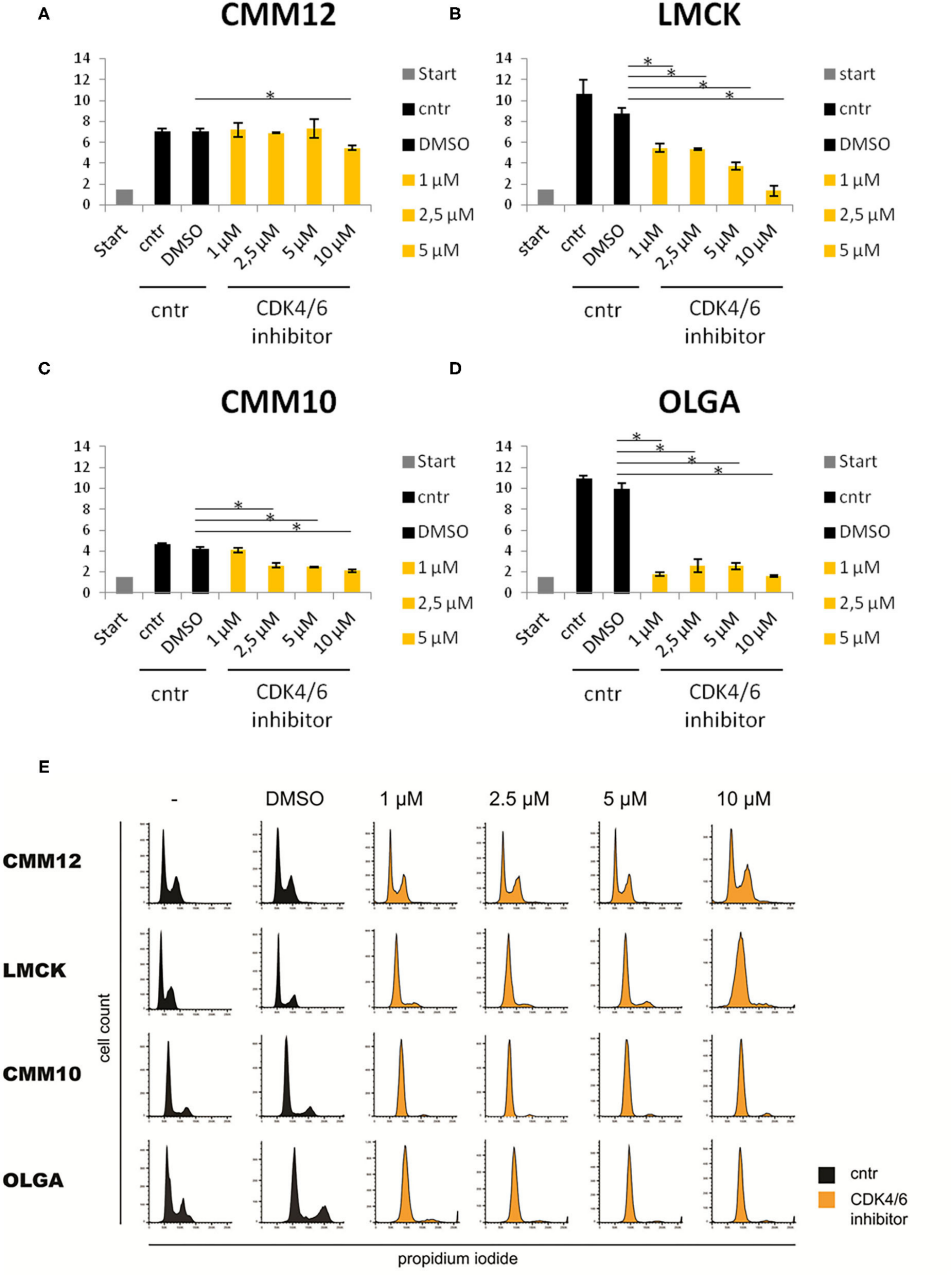
Three of the four tested melanoma cell lines were sensitive to CDK4/6 inhibitor (Palbociclib). Sensitivity of CMM12, CMM10, LMCK, and OLGA melanoma cell lines toward CDK4/6 inhibitor palbociclib. **(A–D)** Cell numbers of melanoma cells treated with different doses of palbociclib for 48 h. Gray bars represent the cell numbers that were seeded at the start of the experiment. Black bars represent the cell numbers of control cells at the end of the experiment. Orange bars represent cell numbers of CDK4/6 inhibitor treated cells at the end of the experiment. Error bars represent the standard error from the mean based on three biological triplicates. *P < 0.05. **(E)** FACS cell cycle analysis of propidium iodide stained melanoma cells treated with 1, 2.5, 5, or 10μM palbociclib, and untreated or 1% DMSO treated controls. Black graphs represent the cell numbers of control cells at the end of the experiment. Orange graphs represent cell numbers of CDK4/6 inhibitor treated cells at the end of the experiment.

Due to the high expression of E2F target genes in canine melanoma tissue samples, we further investigated the possibility of targeting the Rb-E2F pathway, as a new potential anticancer therapeutic approach for canine oral melanoma using a CDK4/6 inhibitor. In LMCK and OLGA cells, a clear and significant reduction in cell numbers was observed after treatment with all concentrations of the CDK4/6 inhibitor (Figures 5A,D). In addition, a minor inhibiting effect of DMSO on cellular proliferation was observed when comparing cell numbers of DMSO-treated control cells to untreated control cells. In CMM10 melanoma cells, the differences between CDK4/6 inhibitor treated cells and control cells were less obvious, although with palbociclib concentrations ≥2.5 μM cell numbers slowly but steadily decreased with increasing concentrations of the compound (Figure 5C). In CMM12 cells, cell numbers remained relatively constant with increasing concentrations of the CDK4/6 inhibitor and a significant reduction of cell proliferation was seen only at the maximum palbociclib concentration tested (Figure 5B; Supplementary Table 4).

FACS cell cycle profiles confirmed the cell number data for CMM12 cells, as cells in all conditions displayed a similar cell cycle profile and no sign of a G1 cell cycle arrest was observed (Figure 5E). In contrast, the cell cycle profiles of palbociclib-treated CMM10, LMCK, and OLGA cells showed a marked decrease in the S-phase population of cells compared to controls, indicating a G1 cell cycle arrest.

### CDK4/6 Inhibitor Decreases E2F Target Gene Expression in Oral Melanoma Cells

**Figure 6:**
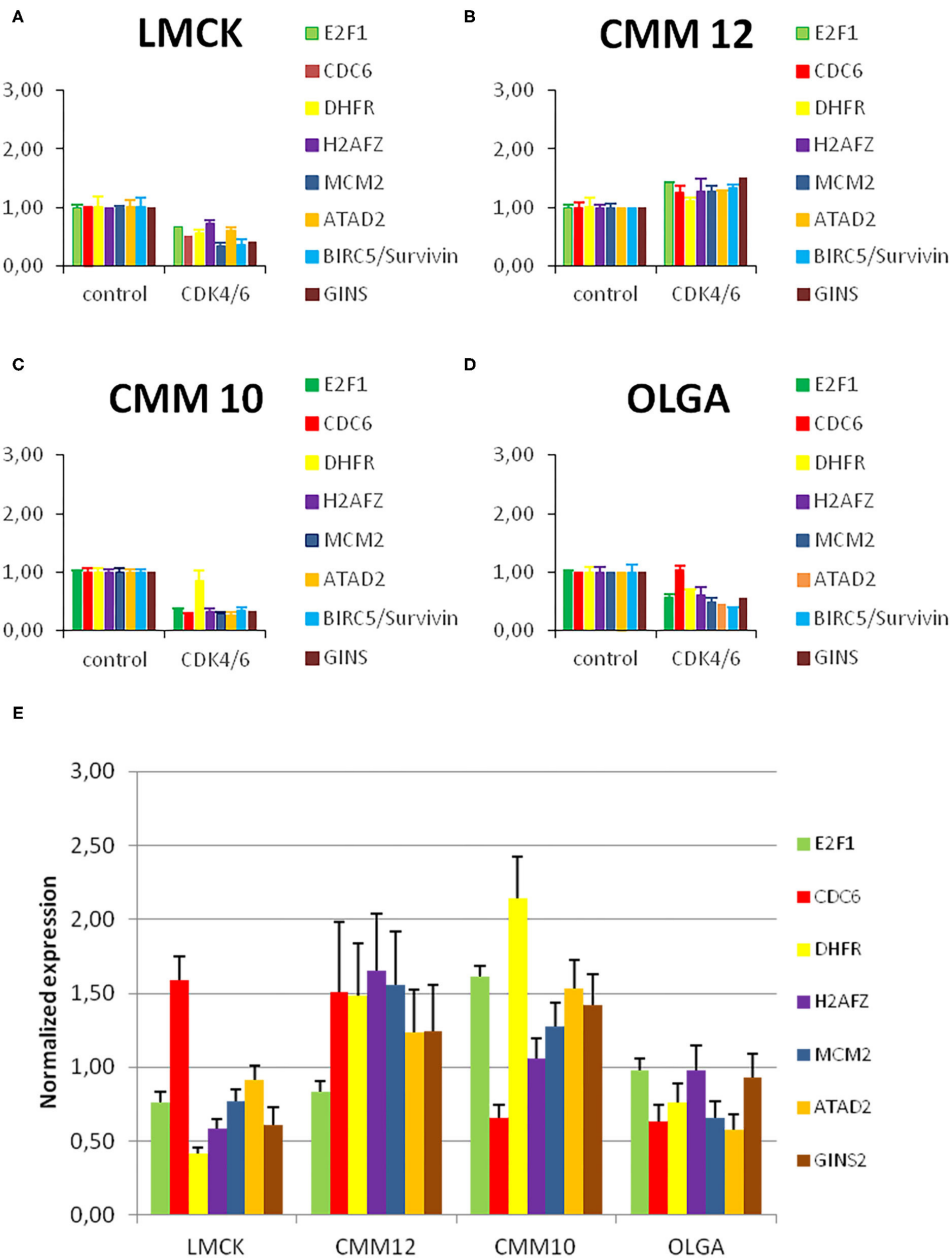
Resistant melanoma cell line showed high levels of H2FZ before and after treatment with CDK4/6 inhibitor. **(A–D)** E2F target gene expression in melanoma cells before and after treatment with a CDK4/6 inhibitor. Normalized E2F target gene transcript levels in melanoma cells treated with 1μM palbociclib compared to untreated control cells. Gene expression was normalized to reference genes GAPDH and RPS5. Bars represent the fold change of the PCR product of indicated genes in CDK4/6 inhibitor treated cells relative to untreated control cells. Error bars represent the standard error from the mean based on two technical replicates and three biological triplicates. **(E)** Normalized E2F target gene transcript levels in melanoma cells under normal conditions compared to the average level of expression of each gene in all cell lines. Gene expression was normalized to reference genes GAPDH and RPS5. Bars represent the fold change of the PCR product of indicated genes in CMM12, OLGA, and LMCK cells relative to the average of all cell lines as a control. Error bars represent the standard error from the mean based on two technical replicates.

In order to explore how the levels of expression of the investigated cell-cycle related genes change during CDK4/6 inhibitor treatment, we analyzed the expression levels of the E2F target genes E2F1, CDC6, DHFR, H2AFZ, MCM2, GINS2, and ATAD2 and compared them between CDK4/6 inhibitor treated cells and controls (Figures 6A–D).

In CMM10, LMCK, and OLGA cells, treatment with 1μM of palbociclib resulted in a reduced expression of the E2F target genes compared to untreated control cells. This reduction was significant in CMM10 for all E2F target genes, with the exception of DHFR; for LMCK it was significant for MCM2 and GINS2. In CMM12 cells, in contrast, the expression of all the genes tended to be slightly higher compared to the control, and only the difference in ATAD2 expression was statistically significant (Supplementary Table 5/1, 5/2). In line with the cell number and FACS data, CDK4/6 inhibitor treatment caused no clear reduction in E2F target gene expression in CMM12 cells.

In order to assess if E2F target gene transcripts expression varies among the different cell lines under normal conditions, we compared the expression of the selected genes in the four melanoma cell lines. Without CDK4/6 inhibitor treatment, in CMM12 cells H2AFZ and MCM2 appeared to be more highly expressed compared to the other melanoma cell lines (Figure 6E).

## Discussion

The results showed in the present work clearly indicate that some E2F target genes, namely H2AFZ and survivin/BIRC5, have potential prognostic value in canine melanocytic tumors. Furthermore, our results demonstrate that pathways regulating the expression of these genes can be targeted to arrest cell proliferation using a CDK4/6 inhibitor. Since resistant cells showed higher levels of expression of some of the same E2F target genes compared to sensitive ones, we suggest that they can be potentially useful for patient selection as predictive markers.

We observed that H2AFZ showed significant differences in its mRNA expression comparing different groups of patients, showing associations with unfavorable outcome (death due to melanoma), increased proliferation rate (mitotic count and Ki67 index), increased hazard of death and developing recurrence/metastasis. H2AFZ together with H2AFV represent two allelic genes encoding for two isoforms of the highly conserved H2A variant H2A.Z, namely H2A.Z.1 and H2A.Z.2, which has a well-established role in transcriptional regulation (43). Histone H2A variants have previously been demonstrated as a biomarker of tumor progression in several types of human cancers (44). In particular, H2A.Z is overexpressed in multiple malignant tumors, such as breast cancer (45), prostate cancer (46), bladder cancer (47), liver cancer (48, 49), as well as malignant melanoma (50, 51). Both H2A.Z isoforms are highly expressed in human melanoma and high expression levels correlate with poor prognosis (52). In line with this data, our results indicate that H2AFZ is an abundantly expressed E2F target gene and a useful prognostic marker to be used in canine melanoma, also in FFPE tissue samples. Results obtained concerning BIRC5, encoding the survivin protein, the smallest member of the IAP (inhibitor of apoptosis) protein family, are in line with previous results we observed in canine cutaneous melanocytic tumors (17), confirming its utility as a prognostic marker in this type of cancer. Besides (wild-type) survivin, BIRC5 encodes four other survivin variants generated through alternative splicing. Survivin is the more intensively studied and widely acknowledged as an orchestrator of cell division and inhibitor of apoptotic pathways (53). Both protein and mRNA survivin expression has been correlated with patient survival in human melanoma (53), in line with our results. The relatively low percentage of samples expressing the gene, however, would suggest that its mRNA levels are not as high as H2AFZ, resulting in several negative FFPE cases. Therefore, we think that this type of marker would be more suitable for fresh tumor samples, instead of formalin fixed cases. All the other investigated genes were detected in a variable percentage of cases and did not show any significant association with clinical data. It would be worth to confirm these results with a larger sample size and performing multivariable analysis to establish the independent prognostic significance of the two markers. Furthermore, considering that the type of surgery applied can deeply impact on the prognosis, further studies recording these data should be performed in order to better understand the prognostic value of the investigated markers.

Based on the high expression of E2F target genes in the investigated tumors, we hypothesized that pathways regulating their expression can be targeted to arrest cell proliferation as a potential new anti-cancer therapy for canine oral melanoma. We selected a CDK4/6 inhibitor, palbociclib, that is currently approved in clinical practice and most advanced in clinical trials (54) and we treated four melanoma cell lines. Palbociclib is a third-generation CDK4/6 inhibitor, orally administered, acting through a competitive and reversible bound to the ATP pocket of the inactive kinase, thus strongly inhibiting both CDK4 and CDK6. As a consequence, RB hyperphosphorilation is prevented, E2F family members are not released, thus the cell will be in a cell cycle arrest phase (55). To our knowledge, this is the first study testing the sensitivity of canine melanoma cell lines to treatment with CDK4/6 inhibitor palbociclib. Using cell number data, FACS cell cycle profiles, and E2F target gene expression profiles, we demonstrated that three of the investigated canine melanoma cell lines, including two primary tumor-derived (CMM10, LMCK) and one metastatic (OLGA) cell lines, were sensitive to the treatment, suggesting a potential use of this drug for the treatment of canine oral melanoma. These data can pave the way for the start of clinical trials in dogs with oral melanoma with a potential important impact for the cure of canine patients as well as for humans with a similar, but extremely rare, form of mucosal melanoma. However, one canine melanoma cell line (CMM12) was resistant to CDK4/6 inhibition of proliferation and G1 cell cycle arrest. In this work, we did not further investigate the mechanisms mediating CDK4/6 inhibitor resistance in CMM12 cells. As a first step in order to understand if the selected E2F target genes could function as predictor markers of CDK4/6 inhibition resistance, we compared the expression profiles of E2F1, CDC6, DHFR, H2AFZ, MCM2, and ATAD2 among the four canine melanoma cell lines. We observed that H2AFZ and MCM2 were expressed at relatively high levels in CMM12 melanoma cells compared to the other cell lines, suggesting a possible utility of E2F target gene expression in tumor cells as markers to predict response to treatment with a CDK4/6 inhibitor. Although further investigations are needed to confirm this hypothesis, we can speculate that increased E2F target gene expression could represent a consequence of Rb mutation, responsible for CDK4/6 inhibitor resistance. Indeed, from previously published works we know that loss of Rb function is the main mechanism that is described to confer resistance to CDK4/6 inhibitors (31). One study showed that Rb immunostaining was detected in most of the canine melanoma cases investigated and associated with a very low or absent p16 immunolabelling (56). However, the applied method could not give any information about the actual functional activity of the detected RB protein in the tissue. In human melanoma cell lines, loss of expression of the endogenous cyclin-dependent kinase inhibitor (CDKN2A), encoding for two distinct potent tumor suppressor proteins, p16^INK4a^ and p14^ARF^, has shown to correlate with sensitivity to palbociclib; while Rb loss correlated with resistance (57). In a mouse model (patient derived xenograft) of human mucosal melanoma carrying CDK4 amplification, a robust anti-tumor effect of palbociclib has been reported (58). Studies on the characterization of the histone variant H2A.Z.2 as a driver of malignant melanoma showed that H2A.Z.2 exerts a key role in promoting cell cycle progression by controlling the transcriptional output of E2F target genes. H2A.Z.2 deficiency sensitizes melanoma cells to chemotherapy and to MEK (also known as mitogen-activated protein kinase (MAPK) kinase or MAP2K) inhibitors, indicating it as an important mediator of cell proliferation and drug sensitivity in malignant melanoma (52). These data, along with our results, strongly suggest that H2AFZ could potentially be used as a predictive marker to estimate melanoma treatment response.

### Conclusions

In the present study, we identified H2AFZ as a novel promising prognostic marker and we highlighted the possible utility of survivin as a prognostic marker also at the mRNA level for both cutaneous and mucosal canine melanocytic tumors. Furthermore, we demonstrated the sensitivity of three oral melanoma cell lines, including a metastatic one, to treatment with a CDK4/6 inhibitor, suggesting this could be a new effective therapeutic approach for canine oral melanoma. Finally, we showed that H2AFZ might be a marker for predicting resistance against CDK4/6 inhibitor treatment.

## Data Availability Statement

The original contributions presented in the study are included in the article/Supplementary Material, further inquiries can be directed to the corresponding author/s.

## Ethics Statement

Ethical review and approval was not required for the animal study because this study involve client-owned animals, retrospectively or prospectively and a client consent of the biopsy material has been collected for each included case. Written informed consent was obtained from the owners for the participation of their animals in this study.

## Author Contributions

LB and AB conceived of the presented idea. AB supervised the findings of this work. LB performed the experiments on tumor tissues and wrote the manuscript. AA performed the *in vitro* experiments. SS performed statistics. CB and IP classified and selected the tumor cases and samples included in the work. All authors discussed the results and contributed to the final manuscript.

## Conflict of Interest

The authors declare that the research was conducted in the absence of any commercial or financial relationships that could be construed as a potential conflict of interest.

## Publisher's Note

All claims expressed in this article are solely those of the authors and do not necessarily represent those of their affiliated organizations, or those of the publisher, the editors and the reviewers. Any product that may be evaluated in this article, or claim that may be made by its manufacturer, is not guaranteed or endorsed by the publisher.
